# Digital Integrated Interventions for Comorbid Depression and Substance Use Disorder: Narrative Review and Content Analysis

**DOI:** 10.2196/67670

**Published:** 2025-05-09

**Authors:** Geneva K Jonathan, Qiuzuo Guo, Heyli Arcese, A Eden Evins, Sabine Wilhelm

**Affiliations:** 1 Center for Digital Mental Health Department of Psychiatry Massachusetts General Hospital Boston, MA United States; 2 Department of Psychological Science University of California Irvine, CA United States; 3 Center for Addiction Medicine Department of Psychiatry Massachusetts General Hospital Boston, MA United States

**Keywords:** digital intervention, depression, depressive, substance use, integrated treatment, evidence-based therapy, comorbidity, substance use disorder, Google Scholar, digital modalities, open coding approach, content analysis, narrative review, literature review, treatment strategies, clinical outcomes, digital health, mental health

## Abstract

**Background:**

Integrated digital interventions for the treatment of comorbid depression and substance use disorder have been developed, and evidence of their effectiveness is mixed.

**Objective:**

This study aimed to explore potential reasons for mixed findings in the literature on integrated digital treatments. We described the methodologies and core characteristics of these interventions, identified the presence of evidence-based treatment strategies, examined patterns across digital modalities, and highlighted areas of overlap as well as critical gaps in the existing evidence base.

**Methods:**

In June 2024, a literature search was conducted in Google Scholar to identify digital integrated interventions for comorbid major depressive disorder and substance use disorder. Articles were included if they described interventions targeting both conditions simultaneously; were grounded in cognitive behavioral therapy, motivational interviewing, or motivational enhancement therapy; and were delivered at least in part via digital modalities. In total, 14 studies meeting these criteria were coded using an open-coding approach to identify intervention characteristics and treatment strategies (n=25). Statistical analyses summarized descriptive statistics to capture the frequency and overlap of these strategies.

**Results:**

Studies included a range of digital modalities: internet (n=6, 43%), computer (n=3, 21%), smartphone (n=2, 14%), and supportive text messaging interventions (n=3, 21%). Half (n=7, 50%) of the studies included participants with mild to moderate depression symptom severity and hazardous substance use. Only 36% (n=5) of the studies required participants to meet full diagnostic criteria for major depressive disorder for inclusion and 21% (n=3) required a substance use disorder diagnosis. Most interventions targeted adults (n=11, 79%), with few targeting young or emerging adults (n=4, 29%), and only 36% (n=5) reported detailed demographic data. Treatment duration averaged 10.3 (SD 6.8) weeks. Internet-based interventions offered the widest range of treatment strategies (mean 11.7), while supportive text messaging used the fewest (mean 4.6). Common treatment strategies included self-monitoring (n=11, 79%), psychoeducation (n=10, 71%), and coping skills (n=9, 64%). Interventions often combined therapeutic strategies, with psychoeducation frequently paired with self-monitoring (n=9, 64%), assessment (n=7, 50%), coping skills (n=7, 50%), decisional balance (n=7, 50%), feedback (n=7, 50%), and goal setting (n=7, 50%).

**Conclusions:**

Among integrated digital interventions for comorbid depression and substance use, there was noteworthy variability in methodology, inclusion criteria, digital modalities, and embedded treatment strategies. Without standardized methods, comparison of the clinical outcomes across studies is challenging. These results emphasize the critical need for future research to adopt standardized approaches to facilitate more accurate comparisons and a clearer understanding of intervention efficacy.

## Introduction

Structured, evidence-based approaches in face-to-face (F2F) settings have proven beneficial for treating major depressive disorder (MDD) and substance use disorder (SUD) [1,2], which frequently co-occur. Researchers are increasingly leveraging digital platforms to enhance the reach and accessibility of these treatments. This shift is particularly relevant given that, among treatment-seeking individuals with MDD, the prevalence of concurrent SUDs ranges from 8.6% to 25%, with lifetime prevalence estimates as high as 42.8% [3,4]. Co-occurring MDD and SUD are associated with heightened symptom severity [5], psychosocial instability, and substantial societal and medical burdens due to disability and health care costs [6-9].

Current treatment guidelines recommend integrated treatment for individuals with co-occurring MDD and SUD to improve treatment outcomes and promote long-term recovery [10]. Cognitive behavioral therapy (CBT), proven effective for both disorders individually in F2F settings [11,12], has been adapted into integrated cognitive behavioral therapy (ICBT) to address the specific challenges of co-occurring illness. ICBT focuses on helping individuals identify and challenge unhelpful thought patterns—such as catastrophizing, black-and-white thinking, and negative self-evaluation—that contribute to both low mood and substance use. In addition, ICBT emphasizes engaging in healthy activities, building a supportive social network, and incorporating motivational interviewing (MI) and motivational enhancement therapy (MET) to encourage behavior change [1,13-16]. Early sessions include clinical assessment, diagnostic feedback, case formulation covering the development and maintenance of the conditions, psychoeducation about the relationship between depression and substance use, and MI to elicit behavior change planning. ICBT addresses maladaptive cognitions by helping individuals identify harmful thought patterns, generate alternative, healthier thoughts, and practice thought-challenging techniques in situations that could potentially lead to substance use relapse or heightened depressive symptoms. It also encourages individuals to plan and schedule activities that promote positive affect and manage pressures that could lead to relapse. Socially, ICBT provides assertiveness and communication training to strengthen social interactions and boost confidence in resisting social pressures to use substances. Finally, ICBT encourages individuals to build coping skills, emergency plans, and relapse prevention strategies.

Despite the availability of ICBT and growing evidence for its effectiveness, most individuals with comorbid MDD and SUD do not receive this recommended treatment [17-19]. Among adults with both conditions, 66% receive substance use treatment, 52% receive mental health treatment, and only 13% receive care for both [20-22]. This siloed approach results in inadequate care [23,24]; some studies show that treating MDD or SUD alone does not increase remission rates of the untreated disorder [25,26]. Many treatment settings lack the resources to meet these individuals’ needs [27]. There is a clear need for accessible, scalable, and effective integrated MDD and SUD treatment programs.

Systematic reviews and meta-analyses have shown that digital interventions can effectively reduce depression severity [28] and substance use [29] when addressed individually. Recent studies suggest that digital integrated treatment may also be effective for co-occurring disorders [30,31]. However, the evidence base for digitally integrated interventions remains limited, with mixed results regarding effectiveness [30,31]. The variability in treatment outcomes among digitally integrated treatments makes it unclear why some interventions succeed in improving depression and substance use outcomes while others fall short.

This review examines digital modalities, methodological approaches, inclusion and exclusion criteria, and evidence-based treatment strategies across existing integrated digital treatments for MDD and SUD. It identifies commonly used strategies, as well as critical gaps and overlaps, to distill effective approaches that can be tailored to specific population needs [32]. In doing so, this analysis aims to highlight treatment strategies that may enhance the efficacy of future digital integrated treatments.

## Methods

### Digital Intervention Identification

In June 2024, the first author (GJ) conducted a literature search in Google Scholar for digital, integrated interventions for MDD and SUD. Eligible articles included one of the terms in Table 1 in the title, abstract, keywords, or article text. The reviewer also screened the reference list of all eligible articles, plus a search of all articles that had cited the included papers.

**Table 1:** Inclusion criteria keywords for digital major depressive and substance use disorders intervention studies.

Category	Term
Digital technology	“digital health,” “digital intervention,” “eHealth,” “mHealth,” “online,” “internet,” “text messages,” “text-messaging,” “mobile,” “mobile phone,” “smartphone,” “smartphone app,” “app,” “computer,” “computer-based”
Depression-related	“major depressive disorder,” “MDD,” “depressive,” “depression,” “depressed mood”
Substance use–related	“substance use disorder,” “SUD,” “alcohol misuse,” “alcohol use disorder,” “hazardous alcohol use,” “alcohol use,” “alcohol abuse,” “cannabis,” “smoking cessation”
Comorbidity	“dual diagnosis,” “comorbidity,” “co-occurring”
Study design	“randomized trial,” “randomized controlled trial,” “clinical trial,” “pilot study”
Intervention or methodology	“intervention,” “program,” “treatment,” “therapy,” “integrated treatment,” “integrated”

### Inclusion and Exclusion Criteria

A report of a digital intervention trial of MDD and SUD treatment was included if (1) the article reported on an intervention intended to improve substance use and depression simultaneously, and encompassed various severity levels from mild symptoms to diagnostic thresholds; this included participants diagnosed with MDD and SUD, as well as those without confirmed diagnoses; (2) the treatment described was grounded in CBT, MI, or MET; (3) the treatment was delivered entirely or partially via digital modality (computer, smartphone, internet, and text message) outside of the traditional F2F therapy setting; and (4) the article was written in English as a peer-reviewed journal article. Studies were excluded if they (1) targeted only depression or only substance use, (2) were published protocols without preliminary outcomes or findings, (3) did not specify theoretical underpinnings or an evidence-based rationale for the treatment, or (4) were based on approaches other than CBT, MI, or MET.

### Identification of Study Characteristics

For each study included in the review (Multimedia Appendix 1), we extracted and summarized inclusion and exclusion criteria, study design, measures used to assess MDD, SUD, treatment adherence, and other outcomes the digital modality used, as well as study duration.

### Treatment Strategies Coding Procedure

Twenty-five treatment strategies (Table 2) were coded in July and August 2024. The content analysis coded any mention of used treatment strategies. The intensity of treatment strategies, specifically the frequency of their presentation within the interventions, was not systematically assessed. In addition, the authors did not independently verify whether these strategies were actually implemented beyond the descriptions provided in the articles and associated supplemental materials. In total, three authors (GJ, QG, and HA) coded all reports (n=14) using an open-coding approach [33]. Initially, we conducted a line-by-line coding of each original article, allowing treatment strategies to emerge directly from the data without using a predefined codebook. This inductive process was followed by team discussions to compare our codes to existing evidence-based treatment strategies [13,17,34-37]. Any disagreements in coding were resolved through consensus among the researchers to confirm that the final coding was consistent with and reflective of the included studies [38].

**Table 2:** Treatment Strategies (n=25) identified in the reviewed apps (n=14).

Treatment strategy	Definition
Activity scheduling	Planning or scheduling activities that counter low mood or substance use (eg, planning and logging daily activities, setting reminders for activities, and tracking activity progress)
Assertiveness training	Teaching individuals to express needs and desires confidently or respectfully (eg, roleplaying communication skills)
Assessment	Assessment of symptoms or behaviors (eg, completing self-report questionnaires like PHQ-9^a^)
Change plans	Creating a structured outline of steps needed to achieve behavioral change, identifying goals to achieve plan, barriers, resources, and strategies to overcome (contingency plans and detailed action steps)
Cognitive restructuring	Identifying and challenging negative thought patterns (eg, reframing and reviewing automatic thoughts)
Coping skills	Teaching strategies to manage mood and cravings
Decisional balance	Weighing the benefits and drawbacks of changing versus maintaining current behaviors (eg, advantages or disadvantages analysis or pros and cons)
Drug refusal skills	Specific content related to effectively refusing substances (eg, interactive scenarios and practice exercises)
Feedback	Providing users with personalized feedback based on their input (self-report questionnaires or assessments), progress, or behavior patterns
Goal setting	Any mention of setting goals or setting specific, measurable, achievable, relevant, and time-bound objectives
Harm reduction	Strategies to reduce negative consequences of substance use
Homework assignments	Tasks for users to complete between sessions
Interpersonal effectiveness	Improving social, communication, and relationship skills
Mindfulness	Exercises to increase present-moment awareness or reduce stress (eg, mindful meditations)
Problem-solving	Developing effective solutions to challenges and obstacles
Psychoeducation	Information about depression, substance use, the relationship between mood and substance use, description of symptoms, connection between thoughts, mood, and behaviors
Relapse prevention	Strategies to sustain progress and prevent relapses (eg, discussion of how to maintain wellness)
Relaxation	Techniques to reduce physical or mental tension (eg, guided progressive muscle relaxation)
Reminders	Alerts to prompt engagement with intervention or health-related activities (eg, daily reminders for the use of intervention, medication, activities, or mindfulness)
Resources	Providing access to additional support or therapeutic options and information outside of intervention
Risk management	Identifying and mitigating potential risks to substance use or mood, learning to recognize situations and emotions that lead to use or depressive episodes, stimulus control, and managing environmental cues that trigger use or negative emotions.
Self-care	Encouraging healthy lifestyle habits to support recovery (eg, exercise, sleep hygiene, and diet)
Self-monitoring	Tracking or logging symptoms, behaviors, progress, daily experiences, and emotions (eg, writing in a diary, mood monitoring, and tracking substance use)
Social supports	Building or maintaining a supportive network (eg, forums or chat features for peer support)
Time management skills	Techniques to organize and prioritize tasks

^a^PHQ-9: Patient Health Questionnaire-9.

### Statistical Analyses

The number, frequency, and percentage of treatment strategies used in the included interventions were summarized using R (version 4.3.2; R Core Team). Plots summarizing the frequencies and overlap of treatment strategies were generated using the “ggplot2” [39] and “pheatmap” [40] packages in R.

## Results

### Intervention Characteristics

Of the 14 studies that met inclusion criteria (Multimedia Appendix 1) [41-55], half of the studies (n=7, 50%) cited a protocol paper with an in-depth description of the research methodology, including participant recruitment procedures, data collection methods, and statistical analysis plans [56-62].

#### Study Design

The majority of studies were randomized controlled trials that compared their integrated digital treatment against treatment as usual (n=5, 36%) [44,47,48,50,55], assessment only condition (n=2, 14%) [42,54], digital health monitoring (eg, HealthWatch; n=1, 7%) [43], or the provision of psychoeducational materials (n=1, 7%) [46].

Some studies compared their integrated treatments against digital mood- or substance-only conditions (n=2, 14%) [44,45].

A minority (n=2, 14%) of studies used an active, in-person comparator, such as F2F integrated treatment or person-centered therapy [41,52], or had no comparator (n=2, 14%; eg, single-arm feasibility trials) [51,53].

#### Eligibility Criteria and Participants’ Characteristics

Only 5 (36%) studies required participants to meet *DSM* (*Diagnostic and Statistical Manual of Mental Disorders*) criteria for MDD, which was confirmed using the Structured Clinical Interview for *DSM-IV* (SCID) [63] or Psychiatric Research Interview for Substance and Mental Disorders (PRISM) [64]. Only three (21%) studies required diagnostic verification of SUD [48-51].

Half (n=7, 50%) of the studies enrolled participants based on the presence of mild to moderate depression symptoms without confirmation of *DSM* criteria [41-47]. These symptoms were assessed via the Beck Depression Inventory-II (BDI-II), Depression, Anxiety, and Stress Scale-21 items, Center for Epidemiologic Studies Depression Scale (CES-D) or Patient Health Questionnaire-9 (PHQ-9) [65-68]. These studies also included participants with problematic or hazardous alcohol or cannabis use, assessed by the Alcohol Use Disorders Identification Test [69] or by how often they used these substances each week. 

Two (14%) studies did not require any formal clinical diagnosis or symptomatology of either depression or substance use, but targeted mood and substance use [53,54].

Most studies (n=11, 79%) evaluated intervention effectiveness among adults (≥18 years). Of these, 4 (29%) studies tested interventions among emerging and young adults (ages 18-35 years) [42,43,46,55]. Three (21%) studies included adolescent participants, with 2 (14%) enrolling individuals aged 16 years and older [41,52], and 1 (7%) enrolling those aged 17 years and older [54]. Only 5 (36%) studies reported detailed demographic information, including race, ethnicity, sexuality, socioeconomic status, and educational background, within their participant samples [42,46,47,53,54].

#### Digital Modality

Regarding the type of technology, the majority were internet-based (n=6, 43%) [42-46,55], followed by offline, computer-based (n=3, 21%) [51,52,70], supportive text messages (n=3, 21%) [48-50,53], and smartphone apps (n=2, 14%) [47,54]. Half were self-guided (n=7, 50%) [42,43,45,47,48,50,53]. The other half involved some level of human support (n=7, 50%) delivered by a licensed psychologist or therapist (n=3) [41,51,52], by supervised doctoral students (n=1) [55], or by trained volunteers (n=1) [54]. Two guided studies did not provide details on the human supporters’ educational background or training qualifications [44,46].

#### Treatment Duration and Adherence

Intervention length ranged from a single session (n=1, 7%) [42], to 4-6 weeks (n=3, 21%) [43,44,54], 8-10 weeks (n=5, 36%) [41,46,47,51,52], and 12-24 weeks (n=5, 36%) [45,48,50,53,55]. Several of the interventions had participants completing multiple modules within a week. On average, the interventions were 10.3 (SD 6.8, median 10, range 0-24) weeks, with text messages having the longest average duration of 20 (SD 6.9) weeks, followed by computer-based (mean 10, SD 0), internet-based (mean 7, SD 4.7), and smartphone apps (mean 6, SD 2.8). Follow-up assessments were conducted for 1 month (n=3, 21%) [42,51,54], 3 months (n=9, 64%) [41,43-45,48,50,52,53,55], 6 months (n=9, 64%) [43-46,48,50,52,53,55], and 12 months (n=3, 21%) [50,52,55]. Most studies (n=11, 79%) included information about the number of sessions or modules completed or the total amount of time the intervention was used; though this metric was not assessed consistently across studies [41,43-47,51-55].

#### Outcome Measurement

Studies reported changes in depression symptom severity using the BDI-II (n=6, 43%) [41,42,48,50,52,66], CES-D (n=4, 29%) [44-46,55], or PHQ-9 (n=3, 21%) [43,51,54]. One (7%) study used the Clinical Outcomes in Routine Evaluation to measure overall changes in psychological health and well-being [53,71].

Almost all studies focused on alcohol as the primary comorbid substance (n=13, 93%) [42-46,48,50,53,55], with some (n=4, 29%) of these also examining problematic cannabis use [41,51,52,54] or tobacco smoking (n=1, 7%) [54].

Substance use outcomes were reported using a variety of measurement tools across studies. The most commonly used instrument was the Timeline Follow-Back (n=6, 43%) [44,46,48,50,51,55,72]. Other measures included the Alcohol Use Disorders Identification Test (n=2, 14%) [45,54,69], the Opiate Treatment Index (n=2, 14%) [41,52,73], the Daily Drinking Questionnaire (n=1, 7%) [42,74], the Rutgers Alcohol Problem Index (n=1, 7%) [42,75], and beverage-specific past week consumption (n=1, 7%) [43]. One (7%) study focused on mental well-being more broadly (distress reduction), so they did not directly assess or report substance use outcomes [53]. Using these measures, studies' primary substance use outcomes were the number of drinks over 7 days (n=6, 43%) [42-46,55], substance use consequences (n=3, 21%) [42,45,46], cumulative abstinence duration (n=2, 14%) [48,50], days to first drink (n=2, 14%) [48,50], and urine toxicology results (n=1, 7%) [51].

### Intervention Content

#### Overview

**Figure 1:**
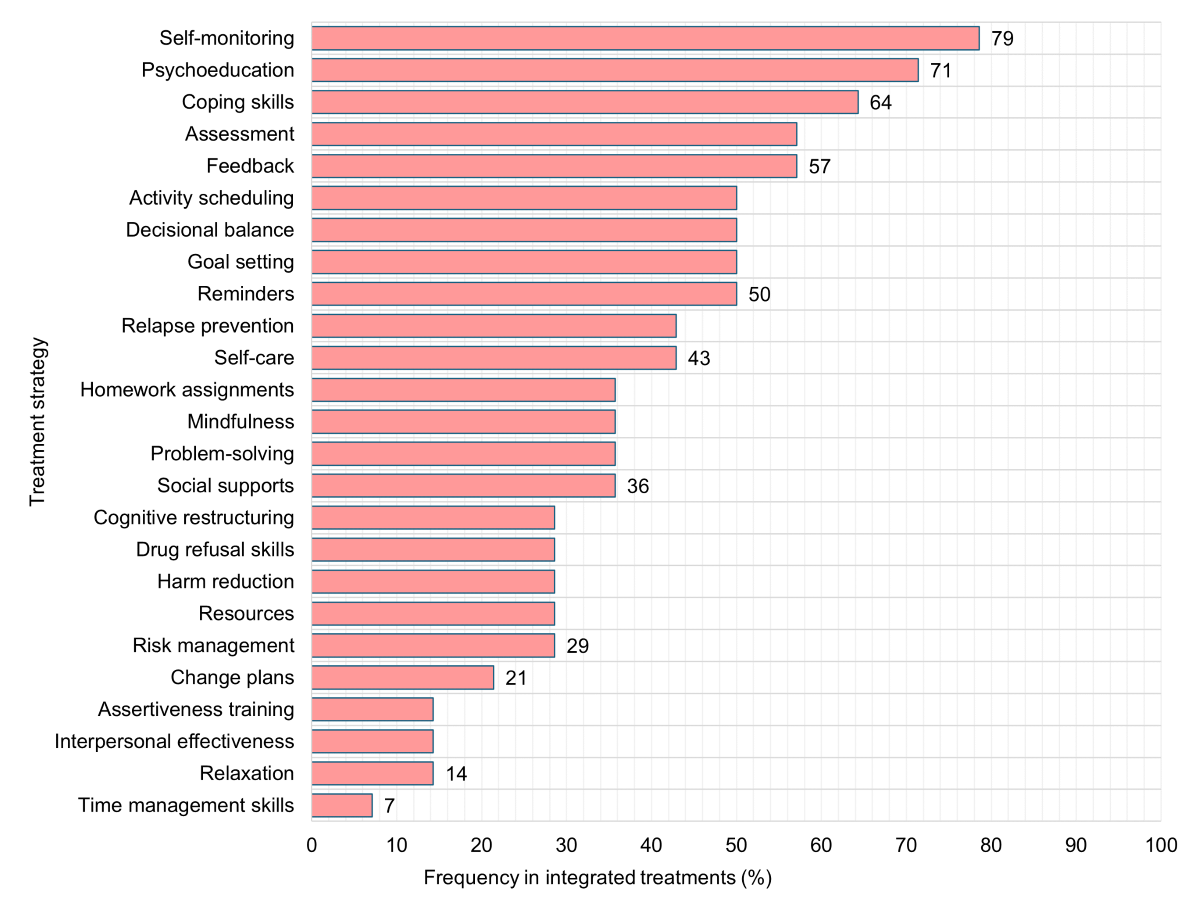
Treatment strategies across integrated digital tools.

All the studies included (n=14, 100%) contained treatment content based on CBT, MI, or MET. Figure 1 shows the frequency of inductively identified treatment strategies (n=25) across all digital treatment modalities.

Fewer than half of the studies (n=5, 36%) reported session-by-session details about the evidence-based treatment strategies offered in the intervention [43,44,47,54,55].

All studies used multiple treatment strategies (mean 9.71, SD 5.08, median 8.5, range 3-19). Most studies used between 5 and 14 treatment strategies. A detailed list of coded strategies in each study can be found in Multimedia Appendix 2. The most frequently used strategies were self-monitoring (n=11, 79%), psychoeducation (n=10, 71%), coping skills (n=9, 64%), feedback and assessment (n=8, 57%). Half of the studies (n=7, 50%) included activity scheduling, decisional balance, goal setting, and reminders. Less common strategies were time management skills (n=1, 7%), relaxation, interpersonal effectiveness, and assertiveness training (n=2, 14%).

**Figure 2:**
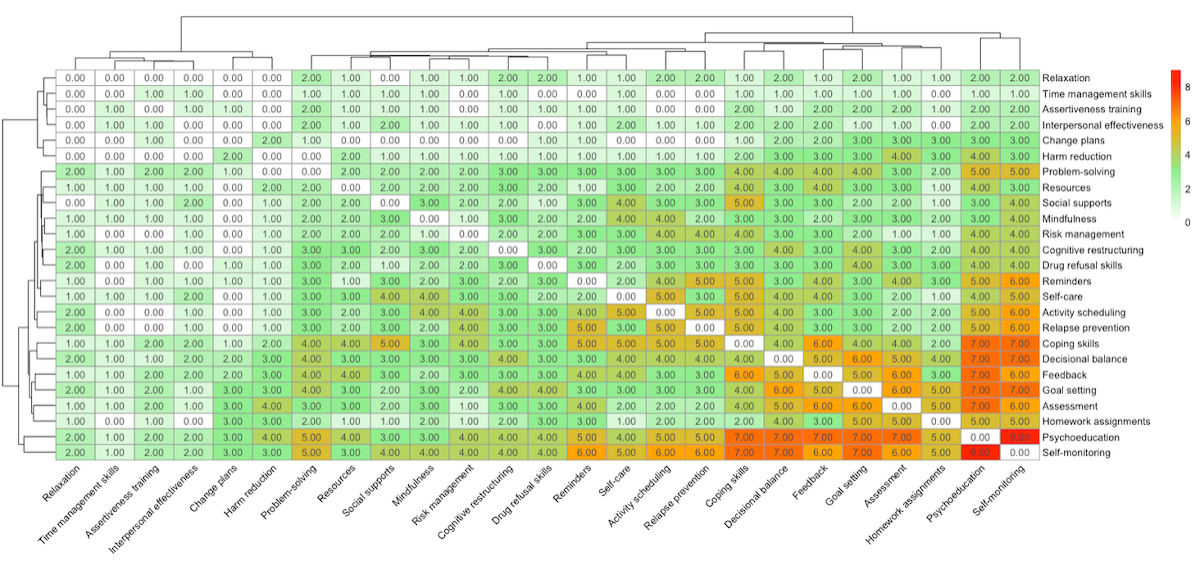
Heatmap of treatment strategy overlaps across digital modalities.

We evaluated the overlap of treatment strategies in the interventions across digital modalities. See Figure 2. Psychoeducation had the highest overlap with other strategies, implemented most frequently in combination with self-monitoring (n=9, 64%), assessment (n=7, 50%), coping skills (n=7, 50%), decisional balance (n=7, 50%), feedback (n=7, 50%), and goal setting (n=7, 50%). Self-monitoring also showed substantial overlap with feedback (n=6, 43%) and coping skills (n=7, 50%). Coping skills were commonly combined with goal setting (n=4, 29%) and feedback (n=6, 43%). Several strategies had minimal overlap with others, including time management skills, assertiveness training, interpersonal effectiveness, relaxation, and mindfulness.

Figure 2 visualizes the overlap of treatment strategies across different interventions. Each row and column represent a treatment strategy, the number within each cell indicates the frequency of overlapping between pairs of treatment strategies across interventions. For example, self-monitoring and psychoeducation are used as strategies in 9 of the 14 studies reviewed. The heatmap uses a color gradient where white and green represent no or minimal overlap and orange or red represents high overlap.

#### Internet-Based Interventions

**Figure 3:**
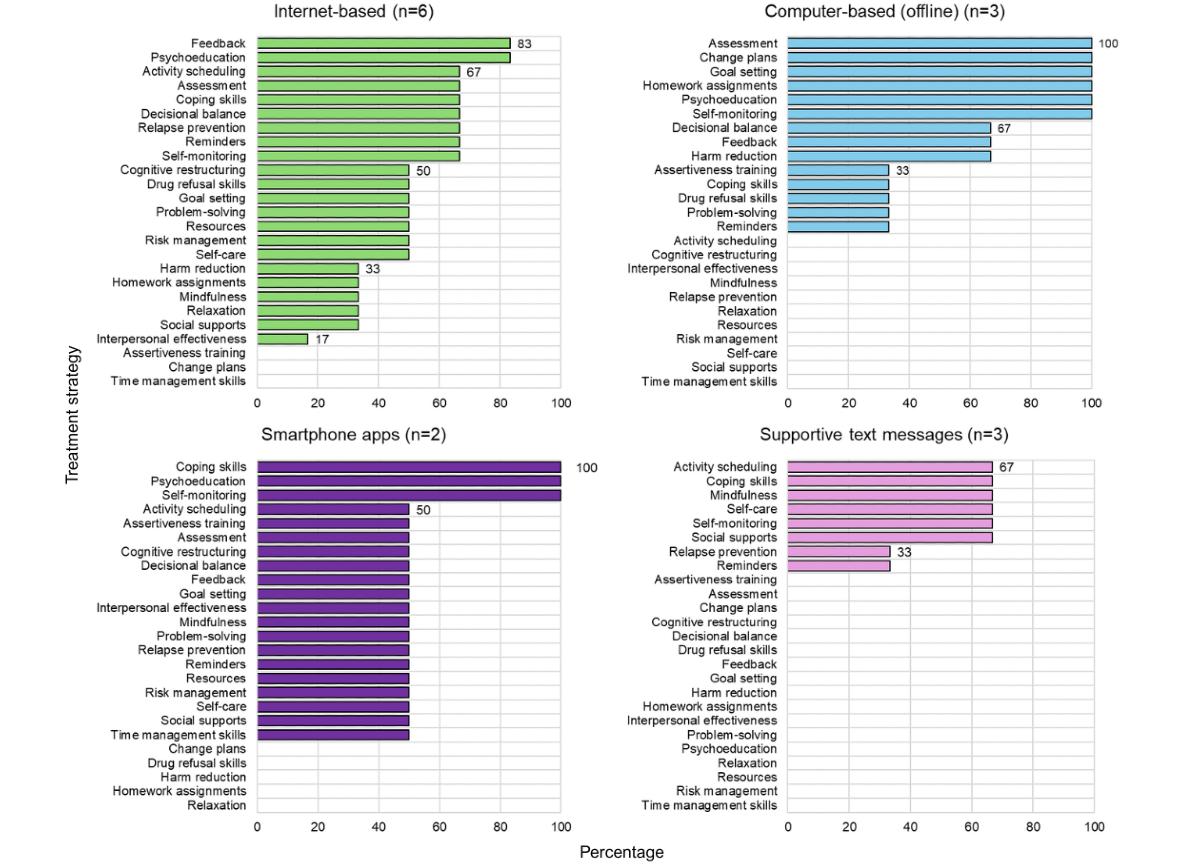
Percentage of treatment strategies by digital modality.

The internet-based interventions (n=6) offered the widest range of treatment strategies (mean 11.7, SD 6.0, median 13.5, range 3-19; Figure 3). A total of 5 of 6 included psychoeducation and feedback. Four included self-monitoring, reminders, relapse prevention skills, decisional balance, coping skills, activity scheduling, and assessment. Only 3 of the 25 identified treatment strategies (12%) were not included in any of the internet-based interventions.

Figure 3 shows the frequency of treatment strategies organized by type of digital modality with the denominator for each graph being the total number of interventions within that digital modality.

#### Computer-Based Interventions

The computer-based interventions (n=3) included an average of 9.7 treatment strategies (SD 2.08, median 9.0, range 8-12; Figure 3). All included self-monitoring, psychoeducation, homework assignments, goal setting, change plans, and self-assessments. Two also included harm reduction skills, feedback, and decisional balance. A larger number of strategies were missing from computer-based interventions: 11 of the 25 strategies (44%) were not included in this modality.

#### Smartphone Apps

The smartphone apps (n=2) included an average of 11.5 (SD 6.36, median 11.5, range 7-15; Figure 3) treatment strategies. Both included self-monitoring, psychoeducation, and coping skills. Additional strategies included activity scheduling, assertiveness training, assessment, cognitive restructuring, decisional balance, feedback, goal setting, interpersonal effectiveness, mindfulness, problem-solving, relapse prevention, reminders, sources, self-care, and time management skills, as well as social support (1/2, 50%). Overall, 5 of the 25 (20%) treatment strategies identified across all interventions were not included in either smartphone app.

#### Supportive Text Messages

The supportive text-messaging interventions (n=3) had the fewest strategies on average, with a mean of 4.6 (SD 0.58, median 5.0, range 4-5; Figure 3). Two of the 3 (67%) interventions included messages related to coping skills, mindfulness, self-care, self-monitoring, and social support. Out of all the digital modalities, supportive text-messaging integrated the fewest treatment strategies, omitting 17 of the 25 (68%) strategies.

## Discussion

### Principal Results

This study aimed to review integrated digital interventions that support individuals with co-occurring MDD and SUD, focusing on study design and characteristics, demographic inclusivity, and therapeutic strategies. Our findings indicate that these interventions often exhibit significant methodological variability, use inconsistent diagnostic criteria, lack demographic diversity, and implement treatment strategies inconsistently. Addressing these issues is critical for making digital integrated interventions more effective and widely applicable for diverse populations with different levels of comorbidity.

### Demographic Diversity and Inclusion Criteria Considerations

Comorbid MDD and SUD vary widely in severity and affect individuals across diverse racial, ethnic, financial, and educational backgrounds. A main finding of our review was the considerable variation in inclusion and exclusion criteria across studies. Most studies targeted individuals with mild to moderate depression and hazardous substance use, with few requiring clinically validated diagnoses. This focus on mild cases and alcohol use is consistent with current F2F treatments, which primarily address depression or anxiety alongside alcohol misuse [18,76,77]. Emerging interest in cannabis misuse [78,79] is beginning to address a broader scope, but significant gaps remain regarding the effectiveness of digital interventions for other substances. While F2F treatments have tested efficacy in clinically diagnosed MDD and SUD [80], the current focus of existing digital interventions on individuals with subclinical or mild-to-moderate symptom severity raises questions about their applicability for those on the more severe end of the comorbidity spectrum.

Demographic data in these studies were generally limited to age and gender, with few studies reporting on racial or socioeconomic diversity. Diverse sample recruitment is critical for advancing equitable mental health care [81]. Known disparities in MDD and SUD treatment access, where some racial groups face unique barriers such as language differences, cultural mistrust, and lack of social support, demonstrate the need for detailed demographic reporting [82]. Without such data, particularly for socially vulnerable communities, assessing whether these interventions are culturally relevant, effective, or capable of providing necessary access is challenging.

Finally, most studies targeted adult populations, with a limited focus on young adults. This is a significant gap, as young adulthood is a formative period where substance use and mood symptoms have the potential to escalate [83,84]. Early intervention for young adults could promote healthier coping mechanisms, resilience, and overall well-being, potentially thwarting the onset of more severe comorbidity later in life and reducing health care burden [85]. Programs for young adults should be tailored to reach individuals at risk or showing early signs of depression or substance use, even if they have not yet been clinically diagnosed.

Future research should expand inclusion criteria to encompass a broader range of MDD and SUD severity, including clinically validated diagnoses, to evaluate these interventions across diverse clinical profiles. It should also expand beyond alcohol to other substances to address gaps in integrated treatments for varying substance use types. Finally, improved demographic reporting is essential to ensure these interventions are accessible, equitable, and effective across diverse racial, ethnic, and socioeconomic backgrounds.

### Methodological Considerations in Assessing Effectiveness

Our review found significant methodological variability across studies, including differences in study design, treatment length, therapist involvement, follow-up periods, outcome measures, and adherence reporting. Study designs varied, with some comparing interventions to active treatments, while others only used assessment or psychoeducation as controls. Treatment duration ranged from a single session to 24 weeks, and therapist involvement also differed, with some interventions being guided and others self-guided. Follow-up periods were inconsistent, from no follow-up to 12 months. Outcome measures varied widely, with depression assessed using tools like the BDI-II and PHQ-9 and substance use measured by tools such as the Timeline Follow-Back with monitoring periods from 7 to 90 days. Adherence reporting was inconsistent as well, with some studies tracking total modules completed and others measuring time spent on the intervention.

Standardizing methodological components would support the identification of effective strategies across diverse populations and clinical settings. For instance, brief smartphone-based interventions may be ideal for community settings or ongoing recovery support, whereas longer, more intensive computer-based programs may better serve individuals requiring intensive, structured care. Standardization would allow for clearer comparisons across studies and more precise matching of intervention tools to treatment needs and stages.

### Variability and Integration of Treatment Strategies Across Digital Modalities

The reviewed interventions consistently included evidence-based strategies grounded in CBT, MI, and MET, though fewer than half provided detailed session-by-session descriptions. The number of strategies used varied widely, with web-based interventions including the most (3 to 19 strategies), followed by smartphone apps, computer-based programs, and supportive text messaging, which used the fewest.

The most frequently used strategies across digital modalities were self-monitoring, psychoeducation, coping skills, feedback, and assessment. About half of the studies incorporated activity scheduling, decisional balance, goal setting, and reminders, while less common strategies included time management, relaxation, interpersonal effectiveness, and assertiveness training. These findings align with existing literature on CBT for MDD and SUD. For instance, a study of CBT experts rating effective components of CBT for depressed adults emphasized the importance of psychoeducation, feedback, and activity scheduling and monitoring [86], reflecting the presence of these strategies in our findings. Similarly, a meta-review of effective interventions for alcohol use disorder identifies feedback, goal setting, and self-monitoring as commonly used strategies, further supporting the frequent use of these strategies in the included studies [87]. However, strategies such as assertiveness training, drug refusal skills, and contingency management, important for SUD treatment [88-90], were less frequently used in the digital interventions we analyzed, highlighting areas for further exploration, especially in addressing the substance use aspect of these integrated treatments.

To our knowledge, this study is the first to map the overlap of integrated digital strategies for MDD and SUD. The heatmap (Figure 2) shows that strategies like psychoeducation often overlap with assessment, goal setting, feedback, and coping skills, covering core elements of ICBT [16]. However, the map also reveals a “scattered” implementation pattern across different modalities. Although CBT is a protocolized treatment, the map demonstrates that there is no standardized approach to integrating these evidence-based strategies, potentially contributing to the variability in treatment outcomes. Some critical strategies for MDD and SUD, such as activity planning (for depression) [91] and drug refusal skills (for substance use) [87], appear less frequently despite being widely recognized as effective in the literature. While future research should explore innovative ways to adapt these more challenging strategies to a digital format, it’s important to acknowledge that some may not fully translate, presenting a persistent limitation of digital approaches. To address this challenge, integrated hybrid models that combine digital interventions with in-person components may offer a more effective solution [92,93].

The scattered distribution also suggests that some interventions may over-rely on strategies that lend themselves well to digital formats (eg, psychoeducation and self-monitoring) while neglecting others that, although potentially more effective, are harder to adapt digitally (eg, practicing drug refusal skills, interpersonal effectiveness, or roleplaying). The nature of some modalities, such as text messaging, inherently limits the strategies that can be implemented. For instance, the simplicity and brevity required for text messaging make it difficult to include more complex strategies, which may require interactive or intensive engagement, such as practicing communication skills or cognitive restructuring. Researchers should recognize that different digital modalities may be suited to different purposes; for example, text messaging may be better suited for ongoing recovery support, while smartphones or internet-based platforms might be more appropriate for delivering more detailed, longer-duration interventions. This understanding should guide the design and evaluation of future digitally integrated interventions, ensuring that each modality is used for its strengths rather than attempting to incorporate all treatment strategies, which could overwhelm the capabilities of certain modalities.

The variability in strategy implementation within digital treatments for MDD and SUD identifies areas for advancement in research and clinical practice. While reflecting the flexibility of digital interventions, these inconsistencies make it difficult to understand whether the issues lie within the digital format itself or the strategies themselves. Future research should focus on testing specific strategies within defined digital modalities to clarify their impact. Comparative studies could, for instance, assess whether strategies like psychoeducation, self-monitoring, and activity planning are more effective on particular platforms (eg, smartphone apps) for specific demographics, such as young adults or those with severe SUD. Identifying the most impactful strategies will help tailor interventions to better meet patient needs based on comorbidity severity.

### Strengths and Limitations

This study reviewed all available digitally integrated treatments for MDD and SUD rather than limiting to only one kind of digital modality. The results offer a broad picture of the present status of digital treatments and their treatment strategies. This review will guide future research and development of these tools by encouraging developers and researchers to be more transparent about the evidence-based strategies incorporated in these interventions, contributing to the effectiveness or lack thereof. This review is limited in that it serves as a descriptive analysis, primarily focused on observing and cataloging the characteristics of the individual treatments and identifying frequencies of treatment strategies used in integrated digital interventions for MDD and SUD. While it provides an idea of the strategies used across different modalities, this study lacks the methodological grounding necessary to draw definitive statistical connections between specific strategies and their effectiveness. As a result, we cannot confidently infer which strategies are most closely associated with successful treatment outcomes or which combinations might be more effective in different contexts. Furthermore, the description of intervention characteristics was based on whether the study provided session-specific details about the implemented evidence-based strategies delivered during the intervention, which may have limited the precision of our treatment coding. Therefore, this study’s findings should be viewed as preliminary, observing trends that warrant further investigation.

### Conclusion

Digital integrated treatments for MDD and SUD have the potential to expand access to evidence-based treatments for individuals with comorbid MDD and SUD. Existing digital integrated treatments offer a wide range of treatment strategies. However, methodological variability in research to date makes summarizing efficacy challenging at this time and highlights study reporting details that will be important for future replicability and generalizability of this literature.

## Appendix

Multimedia Appendix 1 Table of integrated digital treatment programs included in the study.

Multimedia Appendix 2 Presence of treatment strategies across studies (n=14).

## Abbreviations

BDI-II: Beck Depression Inventory-II
CBT: cognitive behavioral therapy
CES-D: Center for Epidemiologic Studies Depression Scale
DSM: Diagnostic and Statistical Manual of Mental Disorders
DSM-IV: Diagnostic and Statistical Manual of Mental Disorders (Fourth Edition)
F2F: face-to-face
ICBT: integrated cognitive behavioral therapy
MDD: major depressive disorder
MET: motivational enhancement therapy
MI: motivational interviewing
MINI: Mini International Neuropsychiatric Interview
PHQ-9: Patient Health Questionnaire-9
SCID: Structured Clinical Interviews for DSM-IV
SUD: substance use disorder
