# Supplementary material for: Digital Integrated Interventions for Comorbid Depression and Substance Use Disorder: Narrative Review and Content Analysis
Source: JMIR Ment Health. 2025 May 9;12:e67670. doi: 10.2196/67670 (PMC12102630; doi:10.2196/67670)
Supplement: Multimedia Appendix 1 [file mental_v12i1e67670_app1.docx]

| Author (year)  [manuscript citation #] | Inclusion and Exclusion Criteria | Sample | Study Design/ Support/Condition | Digital Tx Description | # of Treatment Strategies (%, N =25) | Follow-up (months) | MDD and SUD Outcome | MDD and SUD Results |
| --- | --- | --- | --- | --- | --- | --- | --- | --- |
| **Computer-based (offline)** | | | | | | | | |
| Kay‐Lambkin et al. (2009)^1^  [52] | Inclusion: (1) 16+, (2) mild depression (BDI-II score > 17), (3) lifetime diagnosis of MDD via SCID, (4) problematic alcohol use (> 4 daily drinks for men, >2 for women or weekly cannabis use), (5) absence of TBI, brain disease, cognitive impairment, (6) English-speaking | N=97 46% male Mean age: 35 | 2-arm RCT: computer-delivered vs. therapist-delivered | 1 F2F session, 9 clinician-guided (licensed psychologist), weekly sessions | 32%, 8 | 3, 6, 12 | BDI-II, OTI | - Reduction in depression in CM group over time (P<0.001) w/ gains maintained at 3- and 6 months, followed by stabilization at 12 months (P<0.001)  - Reduction in alcohol consumption in CM group (P<0.001), w/ increase in drinking between 3- and 6 months followed by a reduction at 12 months (P<0.001)  - Reduction in weekly cannabis use in CM group across 12 months (P<0.001)  - Reduction in # of hazardous alcohol in CM group use days across 12-months (P<0.001)  - CM group reductions in all outcome variables comparable to F2F group reductions |
| Kay-Lambkin et al. (2011)^2^  [41] | Inclusion: (1) 16+, (2) mild depression (BDI-II score > 17), (3) harmful alcohol or cannabis use (> 4 daily drinks for men, >2 for women or weekly cannabis use). Exclusion: (1) using cannabis or alcohol below harmful thresholds, (2) under 16 years old, (3) psychotic disorder, (4) non-English speaking, (5) history of TBI | N=274 57% male Mean age: 40 | 3-arm RCT: CAC CBT/MI vs. F2F CBT/MI vs. PCT | 1 F2F session, 9 clinician-guided (intern/licensed psychologists), weekly sessions | 36%, 9 | 3 | BDI-II, OTI | - Reduced depression at 3 months in CBT/MI arms compared to PCT (6.87 vs. 3.84 point reduction, p=0.024), no significant differences between CAC and F2F groups  - Positive correlation between change in depression and change in alcohol use (r = 0.344; P < 0.001), but no association between change in depression and change in cannabis use (r = 0.083, p = 0.179)  - Change in alcohol use was significantly related to tx allocation, with CBT/MI groups associated with four times the reduction in alcohol consumption compared to PCT (mean reduction in drinks/day, 4.88 [CBT/MI] v 1.03 [PCT]; P = 0.004)  -Alcohol use change was linked to changes in depression (r=4.05, P<0.001) and cannabis use (r=0.238, P=0.002). Cannabis use change wasn't significantly different between the CBT/MI groups (P=0.140) despite reductions in both. |
| Glasner et al. (2018)^3^  [51] | Inclusion: (1) 18+, (2) lifetime MDD diagnosis (PRISM) and mild/moderate depression (PHQ-9 score ≥9), (3) cannabis use of ≥ 40 of past 90 days, (5) current antidepressant use, (6) English-speaking at or above 6th-grade level, (7) willingness to examine how depression/cannabis use patterns may relate to one another Exclusion: (1) medical impairment compromising safety, (2) dependent on alcohol or other substances requiring detox, (3) schizophrenia/schizoaffective diagnosis | N= 49 46% male Mean age: 29 | Single-arm trial, computer-based | 1 F2F session, 9 clinician-guided (licensed therapist), weekly sessions | 48% 12 | 1 | PHQ-9, TLFB | - Depression reduced from moderately severe (M=13.3, SD=4.7) to mild at week 10 (M=8.0, SD=5.3), p<0.001, D=0.89), at 1-month follow up severity further reduced (M=6.0, SD=4.9; P<0.001, d=1.52)  - Percentage of days in past 30 w/ reported cannabis use significantly (p=0.05, d=0.54) declined from baseline (M=69%, SD=29%) to EOT (M=52%, SD=34%)  - # of times cannabis was used per day on use days declined significantly from M=2.7 (SD=1.6) to M=1.7 times (SD=0.9) at discharge (P<0.05, d=0.79) |
| **Internet-based interventions** | | | | | | | | |
| Geisner et al. (2015)^4^  [42] | Inclusion: (1) 18-24, (2) mild depression (BDI-II score ≥ 14), (3) hazardous drinking (AUDIT score ≥ 8), (4) recent 4+/5+ drinks (women/men) at least once in the past month, (5) enrolled as Pacific Northwestern student | N=311 38% male Mean age: 20 | 4-arm RCT: online alcohol tx vs. online mood tx, vs. online integrated tx, vs. assessment only | Single time-point self-guided tx, w/ 5-week follow-up to review feedback | 24%, 6 | 1 | BDI-II, DDQ, RAPI | - No significant differences between tx conditions on depression (P=0.78), typical weekly drinking (P=0.54), or alcohol consequences (P=0.28)  - Baseline depression scores influenced the tx effect on alcohol-related problems at follow-up, controlling for gender, race, and baseline alcohol-related problems (P=0.01)  - Individuals with lower baseline depression in the alcohol-only group and integrated group reported significantly fewer alcohol-related problems at follow-up compared to control (9.32 vs. 10.16 vs. 16.51, P=0.01) |
| Deady et al. (2016)^5^  [43] | Inclusion: (1) 18-25, (2) moderate depression (DASS-21 score ≥ 7), (3) hazardous drinking (AUDIT score > 8), (4) Internet access, (5) Australian resident Exclusion: (1) psychosis, (2) non-English speaking, (3) acute suicide risk in past 2 weeks, (4) daily cannabis/weekly amphetamine use | N=104 40% male Mean age: 22 | 2-arm RCT: Online tx vs. control (HealthWatch) | 4 self-guided modules over 4 weeks | 56%, 14 | 3, 6 | PHQ-9, TOT-AL | - Tx group had significant reduction in depression severity at EOT (95% CI -8.18 to -3.70; P<0.001, d=1.09), 3- (95% CI -2.52 to 2.53; P<.99) and 6-months (95% CI -1.38 to 4.57; P=0.29)  - No significant difference in depression between groups at 3- (95% CI -5.10 to 2.90; P=0.59; d=0.15) or 6 months (95% CI -6.45 to 2.29; P=.35; d=0.39).  - Tx group demonstrated a significant reduction in drinks per week from baseline to EOT (95% CI 0.32-0.65; P<.001, d=1.07) and 3-months (95% CI -0.46-1.51, p=.55)  - Between 3- and 6 months # of drinks per week in tx group was still lower than baseline (95% CI 0.96-1.90; P=0.04)  - Compared to control, tx group had significant reduction in drinking days per week at EOT (95% CI; P<0.001, d=1.06), with 79% greater reduction in drinking days (95% CI1.22-2.64; P=0.003), this persisted to 3- (95% CI 0.70-1.47; p=.94) and 6-month follow up (95% CI 0.93-1.84). |
| Baumgartner et al. (2021)^6^  [44] | Inclusion: (1) 18+, (2) moderate depression (CES-D score ≥ 10), (3) hazardous drinking (AUDIT score ≥ 8), (4) regular internet access, (5) German speaking. Exclusion: (1) Current psychosocial/pharmacological treatment for alcohol use or depression, (2) recent (past 30 days) opioid, cocaine, or amphetamines use, (3) frequent (> 3 times weekly in past 30 days) cannabis use, (4) hx of treatment for cardiovascular problems, (5) past suicidal ideations or plans, (6) pregnancy or breastfeeding (for women). | N=689 52% male Mean age: 43 | 3-arm RCT: integrated vs. alcohol only vs. control (IAU) | 8 coach-guided (training/education-level unspecified) modules over 6 weeks | 52%, 13 | 3, 6 | CES-D, TLFB | - At 3 and 6 months, both txs had significantly greater reductions in alcohol consumption than control, at 3 months, integrated tx reductions were 17.4 (SD=23.96) standard drinks, alcohol only tx was 20.9 (SD=23.38) compared to 14.2 (SD=32.8) in controls (P<0.001, d=0.24); reduced drinking persisted through 6 months for active tx vs. controls (P=0.004, d=0.20)  - No evidence for superior effect of integrated tx over AUD-only tx at 3 months (P=0.206) or 6 months (P=0.366)  - At 6 months, integrated and alcohol-only tx had greater depression reductions than control (6.6 [SD=9.3] vs. 7.3 [SD=11.6] vs. 2.6 [SD=10]), but there was no significant difference between tx groups (P=0.890)  - At 6 months, the alcohol-only group reported significantly fewer consumption days over the previous 7 days, including an average decrease of 1.9 days (SD=2.4) compared to controls 0.9 days (SD=2.3; P=0.005, d=0.42) |
| Cunningham et al. (2021)^7^  [45] | Inclusion: (1) 18+, (2) moderate depression (PHQ 9 score ≥ 10), (3) hazardous alcohol use (AUDIT score ≥ 8) Exclusion: (1) current suicidal ideation (PHQ-9 item 9 score >0, due to recruitment issues later changed to >1), (2) one person per household | N=988 27% male Mean age: 36 | 2-arm RCT: online mood tx vs. online integrated tx | 5 self-guided modules – 29 activities over 12 weeks | 12%, 3 | 3, 6 | CES-D, # of drinks per week, AUDIT-C | - Significant reductions in depression ratings at 3 and 6 months (P < 0.001) for both tx groups, with no superiority for integrated tx (P = 0.787)  - Significant reductions in the # of drinks reported at 3 and 6 months (P < .001) for both tx groups –no superiority for integrated tx (P = 0.374) |
| Frohlich et al. (2021)^8^  [46] | Inclusion: (1) 18-35, (2) moderate depression (CES-D score >16) and/or moderate anxiety (GAD=7 score >10), (3) hazardous alcohol use (AUDIT-C score >3 females, >4 males), (4) English speaking, (5) internet access Exclusion: (1) current treatment for alcohol misuse and/or depression/anxiety, (2) more than minimal risk on suicidality screener (P4), (3) current psychosis or mania | N=222 32% male Mean age: 25 | 2-arm RCT: integrated online vs. control (psychoeducation) | 12 research assistant-guided (training /education unspecified) modules over 8 weeks (1-2 per week) | 60%, 15 | 6 | CES-D, TLFB | - Significant time by condition interaction (P= 0.036) where tx group showed larger reductions in depression across tx (P < 0.001) compared to control (P = 0.012)  - At week 8, no significant difference in total weekly alcohol consumption between tx and control (p = 0.219) but a significant reduction in hazardous drinking (p = 0.024) in tx group  - Tx group showed larger reductions in hazardous drinking (P< .001) compared to control (P = 0.001)  - At 6 months, tx group continued to show larger reductions in hazardous drinking compared to control (P =0.026) |
| Schouten et al. (2024)^9^  [55] | Inclusion: (1) 18-35, (2) self-reported or medical record of depression diagnosis and enrolling in or currently in F2F treatment for depression, (3) hazardous alcohol use (AUDIT ≥ 8 men, ≥ 5 women), (4) Dutch speaking, (5) contact information, (6) healthcare insurance, (7) computer/mobile device access, (8) written informed consent. Exclusion: (1) acute psychosis, (2) primary diagnosis of severe AUD, (3) dementia, (4) waitlisted for inpatient mental health care, (5) pregnancy | N=163 22% male Mean age: 25 | 2-arm RCT: internet-based tx vs. TAU | 6 coach-supported (Ph.D. student/ supervised research assistant) modules, recommended 1 module per 2 weeks (avg. ~12 weeks) | 76%, 19 | 3, 6, 12 | CES-D, TLFB | - No significant differences in depression between the tx group and control at 3-month (P = 0.732) and 6-month follow-ups (P = 0.793)  - Significant reduction in # of weekly alcoholic drinks in tx group compared to control at 3-month (P =0.009) and 6-month follow-ups (P = 0.032) |
| **Smartphone apps** | | | | | | | | |
| Dahne et al. (2023)^10^  [47] | Inclusion: (1) 18+, (2) mild depression (PHQ-8 score ≥10), (3) currently smoking ≥5 cigarettes/day for ≥ 25 of the last 30 days, for ≥ last 6 months, (4) seen by a primary care provider in the last year, (5) English-speaking.  Exclusion: (1) reported suicidality at baseline, (2) severe visual impairment limiting app use, (3) contraindications for nicotine replacement therapy | N=164 47% male Mean age: 38 | 2-arm RCT: smartphone app vs. TAU | Self-guided use for 8 weeks, 2 weeks of NRT in tx group | 28%, 7 | N/A | BDI-II, # of cigarettes per day (CPD) | - Both groups showed a decrease in depression over time (M= 8.57, SE = 0.86 points from week 1 to week 12; P <0.001), but tx group reported lower depression over tx duration compared to TAU (M= 3.72, SE = 1.37 points less; P=0.01)  - Regarding abstinence, tx group reported significantly higher floating and 7-day PPA relative to control at week 4 (week 4 (Floating: 14% vs. 0%; P=0.01 and 7-day PPA: 11% vs. 0%; P=.02), week 8 (Floating: 19% vs. 2%; P=0.01 and 7-day PPA: 12% vs. 0%; P=0.02), and week 12 (Floating: 24% vs. 4%; P=0.003 and 7-day PPA: 16% vs. 2%; P=0.02)  - Both groups decreased their CPD throughout tx (M= 4.71, SE=0.42 CPD less by week 12; P<.001), but tx group reported smoking less CPD than TAU group (difference of mean 1.97, SE 0.93 CPD less; P=.03). |
| Vereschagin et al (2024)^11^  [54] | Inclusion: (1) 17+, (2) access to smartphone w/ WIFI or cellular data, (3) English-speaking, (4) UBC Vancouver student Exclusion: (1) current suicidal plan (assessed via a screening question – Do you have a current suicidal plan?) | N=1489 37% male Mean age: 20 | 2-arm RCT: smartphone app vs. assessment only | Peer coach (trained university student volunteer) guided 30 days of check-ins | 64%, 16 | 2-week, 1 month | PHQ-9, USAUDIT-C | - Tx group had significantly greater average reductions in depression compared to control (95% CI −1.08 to −0.17; P=0.007; d=−0.11)  - Reduction in alcohol use among tx group observed but not significant (P=0.23)  -Tx group had a significant 20% reduction in their frequency of cannabis use (P=0.03) |
| **Supportive text messages** | | | | | | | | |
| Agyapong et al. (2012, 2013)^12, 13^  [48,49] | Inclusion: (1) 18+, (2) diagnosis of MDD and Alcohol Dependency Syndrome/Alcohol Abuse (SCID), (3) no cognitive deficits (MMSE score ≥25), (4) able to provide informed consent, (5) enrolled in inpatient dual diagnosis treatment program in St Patrick’s University Hospital, (5) owns a mobile phone, (6) familiar with text messaging, (7) able to read.  Exclusion: (1) bipolar disorder (SCID), (2) psychotic disorder (SCID), (3) current polysubstance use (SCID) | N=54 46% male Mean age: 49 | 2-arm RCT: texts w/ integrated content vs. TAU | 2 daily text messages for 3 months (self-guided) | 20%, 5 | 3, 6 | BDI-II, TLFB | - Significant difference in 3-month depression between tx group and control, mean difference in change was -7.9 (95% CI -13.06 to -2.76, P = 0.003, d = 0.85).  - Trend for greater CAD in tx group vs. control 88.3 (SD =6.2) vs. 79.3 (SD=24.1), (P=0.08, d=0.51)  - At 6 months, no significant difference in depression between tx group and control 13.28 (SD=8.7) vs. 15.08 (SD=11.37; P=0.66) or CAD 84.14 days (SD=9.20) vs. 74.73 (SD=28.97; P=0.16)  - At 6 months, significantly higher days to first drink in tx group vs. control 119.9 (SD=47.7) vs. 62.4 (SD=44.9; P =0.01) |
| O’Reilly et al. (2019)^14^  [50] | Inclusion: (1) 18-70, (2) current MDE (SCID) and mild depression (BDI-II score ≥ 14), (3) alcohol dependence (SCID), (4) no cognitive deficits (MMSE score ≥25), (5) completed inpatient program, (5) owns mobile phone Exclusion: (1) other psychiatric conditions such as psychosis | N=95 46% male Mean age: 48 | 2-arm RCT: texts w/ integrated content + TAU vs. TAU | 2 daily text messages over 6 months (self-guided) | 15%, 4 | 3, 6, 12 | BDI-II, TLFB | - Depression significantly reduced at 3 months in tx group compared to control (P=0.02)  - Between baseline and 3-month tx time point, there was no significant difference between groups in number of days drinking (P=0.38)  - Tx group showed significantly greater reduction in units per drinking day from baseline to 6 months compared to control (P=0.03)  - At 6-months, no significant difference was found between groups in drinking or depression change scores (P=0.78) |
| Noble et al. (2021)^15^  [53] | Inclusion*: (1) 18+, (2) mobile phone with text-messaging capabilities, (3) familiarity with text messaging, (4) ability to read English  *Patients from this study were recruited from mental health/addiction services clinic – though clinical relevancy was not required for inclusion/exclusion | N=296 37% male Mean age: 36 | Single arm RS: texts w/ integrated content | One daily text message for 6 months (self-guided) | 20%, 5 | 3, 6 | CORE-10 | -Improvement of 22.79% in anxiety, depression, physical problems, and risk to self from baseline to 12 weeks (mean difference −5.71;95% CI = 2.03–2.74; P=<0.001, d=0.89), and overall improvement (baseline to 6-months; P=0.02, d=0.64) of 18.97% with a mean difference of −4.58 (95% CI = 2.85–3.57)  -There was no statistically significant change in the CORE-10 scores from 12-weeks to 6-months (P=0.212, d=-0.36)  -Most participants sought help for mental health concerns (79% at 12 weeks, 75% at 6 months), a smaller percentage for substance use concerns (9% at 12 weeks, 14% at 6 months) |

AUD: alcohol use disorder, AUDIT: Alcohol Use Disorders Identification Test (AUDIT)^16^, BDI-II: Beck Depression Inventory-II^17^, BtB: Better than Booze, CAC: clinician-assisted computerized treatment, CAD: cumulative abstinence duration, CBT: cognitive behavioral therapy, CES-D: Centre of Epidemiologic Studies of Depression Scale^18^, CORE-10: Clinical outcomes routine evaluation system^19^, CPD: cigarettes per day, CAD: Cumulative Abstinence Duration, CM: computer condition, CPD: cigarettes per day, CUD: cannabis use disorder, CUDIT: Cannabis Use Disorders Identification Test^20^, CYD: Check Your Drinking, DASS-21: Depression, Anxiety Stress Scales – 21 items^21^, DDQ: Daily Drinking Questionnaire^22^, DSM: Diagnostic and Statistical Manual of Mental Disorders^23^, EOT: end of treatment, F2F: face-to-face, GAD-7: generalized anxiety disorder 7-item scale)^24^, HX: history, IAU: Internet access as usual, MDD: Major depressive disorder, MDE: Major depressive episode, MET: motivational enhancement therapy^25^, MMSE: Mini-Mental State Examination^26^, MI: motivational interviewing, NR: not reported, NRT: Nicotine replacement therapy^27^, OTI: opiate treatment index^28^, P4: P4 suicidality screener^29^, PCT: person-centered therapy^30^, PHQ-8: Patient Health Questionnaire-8 (does not include suicide item)^31^, PHQ-9: Patient Health Questionnaire-9 (includes suicide item)^32^; PPA: point prevalence abstinence, RAPI: Rutgers Alcohol Problem Index^33^, RCT: randomized controlled trial, RS: retrospective study, SCID: structured clinical interview for DSM-IV^34^, TAU: treatment as usual, TBI: traumatic brain injury, TLFB: timeline follow back^35^, TOT-AL: beverage-specific past week alcohol consumption^36^, TX: treatment

References

1. Kay‐Lambkin, F.J., et al., *Computer‐based psychological treatment for comorbid depression and problematic alcohol and/or cannabis use: a randomized controlled trial of clinical efficacy.* Addiction, 2009. **104**(3): p. 378-388.

2. Kay-Lambkin, F., et al., *Acceptability of a clinician-assisted computerized psychological intervention for comorbid mental health and substance use problems: treatment adherence data from a randomized controlled trial.* Journal of Medical Internet Research, 2011. **13**(1): p. e1522.

3. Glasner, S., et al., *Preliminary outcomes of a computerized CBT/MET intervention for depressed cannabis users in psychiatry care.* Cannabis (Research Society on Marijuana), 2018. **1**(2): p. 36.

4. Geisner, I.M., et al., *Brief web-based intervention for college students with comorbid risky alcohol use and depressed mood: does it work and for whom?* Addictive behaviors, 2015. **42**: p. 36-43.

5. Deady, M., et al., *An online intervention for co-occurring depression and problematic alcohol use in young people: primary outcomes from a randomized controlled trial.* Journal of medical Internet research, 2016. **18**(3): p. e5178.

6. Baumgartner, C., et al., *“Take Care of You”–Efficacy of integrated, minimal-guidance, internet-based self-help for reducing co-occurring alcohol misuse and depression symptoms in adults: Results of a three-arm randomized controlled trial.* Drug and alcohol dependence, 2021. **225**: p. 108806.

7. Cunningham, J.A., et al., *Randomized controlled trial of online interventions for co-occurring depression and hazardous alcohol consumption: primary outcome results.* Internet Interventions, 2021. **26**: p. 100477.

8. Frohlich, J.R., et al., *Efficacy of a minimally guided internet treatment for alcohol misuse and emotional problems in young adults: Results of a randomized controlled trial.* Addictive behaviors reports, 2021. **14**: p. 100390.

9. Schouten, M.J., et al., *Effectiveness of a digital alcohol intervention as an add-on to depression treatment for young adults: results of a pragmatic randomized controlled trial.* Psychological Medicine, 2024: p. 1-12.

10. Dahne, J., et al., *Behavioral Activation–Based Digital Smoking Cessation Intervention for Individuals With Depressive Symptoms: Randomized Clinical Trial.* Journal of Medical Internet Research, 2023. **25**: p. e49809.

11. Vereschagin, M., et al., *Effectiveness of the Minder mobile mental health and substance use intervention for university students: randomized controlled trial.* Journal of medical internet research, 2024. **26**: p. e54287.

12. Agyapong, V.I., et al., *Supportive text messaging for depression and comorbid alcohol use disorder: single-blind randomised trial.* Journal of affective disorders, 2012. **141**(2-3): p. 168-176.

13. Agyapong, V.I., D.M. McLoughlin, and C.K. Farren, *Six-months outcomes of a randomised trial of supportive text messaging for depression and comorbid alcohol use disorder.* Journal of affective disorders, 2013. **151**(1): p. 100-104.

14. O’Reilly, H., et al., *Alcohol use disorder and comorbid depression: a randomized controlled trial investigating the effectiveness of supportive text messages in aiding recovery.* Alcohol and alcoholism, 2019. **54**(5): p. 551-558.

15. Noble, J.M., et al., *Text4Support mobile-based programming for individuals accessing addictions and mental health services—retroactive program analysis at baseline, 12 weeks, and 6 months.* Frontiers in psychiatry, 2021. **12**: p. 640795.

16. Reinert, D.F. and J.P. Allen, *The alcohol use disorders identification test (AUDIT): a review of recent research.* Alcoholism: Clinical and Experimental Research, 2002. **26**(2): p. 272-279.

17. Beck, A.T., R.A. Steer, and G. Brown, *Beck depression inventory–II.* Psychological assessment, 1996.

18. Eaton, W.W., et al., *Center for epidemiologic studies depression scale: Review and revision.* The use of psychological testing for treatment planning and outcomes assessment, 2004.

19. Barkham, M., et al., *The CORE‐10: A short measure of psychological distress for routine use in the psychological therapies.* Counselling and Psychotherapy Research, 2013. **13**(1): p. 3-13.

20. Adamson, S.J. and J.D. Sellman, *A prototype screening instrument for cannabis use disorder: the Cannabis Use Disorders Identification Test (CUDIT) in an alcohol-dependent clinical sample.* Drug and alcohol review, 2003. **22**(3): p. 309-315.

21. Henry, J.D. and J.R.J.B.j.o.c.p. Crawford, *The short‐form version of the Depression Anxiety Stress Scales (DASS‐21): Construct validity and normative data in a large non‐clinical sample.* 2005. **44**(2): p. 227-239.

22. Collins, R.L., G.A. Parks, and G.A. Marlatt, *Social determinants of alcohol consumption: the effects of social interaction and model status on the self-administration of alcohol.* Journal of consulting and clinical psychology, 1985. **53**(2): p. 189.

23. Association, A.P. and A.P. Association, *Diagnostic and statistical manual of mental disorders: DSM-5*. 2013, United States.

24. Spitzer, R.L., et al., *A brief measure for assessing generalized anxiety disorder: the GAD-7.* Archives of internal medicine, 2006. **166**(10): p. 1092-1097.

25. Miller, W.R. and S. Rollnick, *Motivational interviewing: Helping people change*. 2012: Guilford press.

26. Cockrell, J.R. and M.F. Folstein, *Mini-mental state examination.* Principles and practice of geriatric psychiatry, 2002: p. 140-141.

27. Molyneux, A., *Nicotine replacement therapy.* Bmj, 2004. **328**(7437): p. 454-456.

28. Darke, S., et al., *Development and validation of a multidimensional instrument for assessing outcome of treatment among opiate users: the Opiate Treatment Index.* British journal of addiction, 1992. **87**(5): p. 733-742.

29. Dube, P., et al., *The p4 screener: evaluation of a brief measure for assessing potential suicide risk in 2 randomized effectiveness trials of primary care and oncology patients.* The Primary Care Companion for CNS Disorders, 2010. **12**(6): p. 27151.

30. Raskin, N.J. and C.R. Rogers, *Person-centered therapy.* 2005.

31. Kroenke, K., et al., *The PHQ-8 as a measure of current depression in the general population.* Journal of affective disorders, 2009. **114**(1-3): p. 163-173.

32. Kroenke, K., R. Spitzer, and J. Williams, *The patient health questionnaire (phq-9)–overview.* J. Gen. Intern. Med, 2001. **16**: p. 606-616.

33. Earleywine, M., J.W. LaBrie, and E.R. Pedersen, *A brief Rutgers Alcohol Problem Index with less potential for bias.* Addictive behaviors, 2008. **33**(9): p. 1249-1253.

34. First, M.B., *Structured clinical interview for the DSM (SCID).* The encyclopedia of clinical psychology, 2014: p. 1-6.

35. Sobell, L.C. and M.B. Sobell, *Timeline follow-back: A technique for assessing self-reported alcohol consumption*, in *Measuring alcohol consumption: Psychosocial and biochemical methods*. 1992, Springer. p. 41-72.

36. Khadjesari, Z., et al., *Test–retest reliability of an online measure of past week alcohol consumption (the TOT-AL), and comparison with face-to-face interview.* Addictive behaviors, 2009. **34**(4): p. 337-342.
