# Supplementary material for: Digital Integrated Interventions for Comorbid Depression and Substance Use Disorder: Narrative Review and Content Analysis
Source: JMIR Ment Health. 2025 May 9;12:e67670. doi: 10.2196/67670 (PMC12102630; doi:10.2196/67670)
Supplement: Multimedia Appendix 2 [file mental_v12i1e67670_app2.docx]

**Multimedia Appendix 2.** Presence of treatment strategies across studies (n=14).

Each row represents an evidence-based strategy (e.g., “Activity scheduling,” “Assertiveness training,” “Assessment”), and each column represents one of the 14 studies included in the analysis, organized by digital modality. A filled in square indicates that the strategy was present in the study (coded as 1), and blank spaces indicate absence (coded as 0).


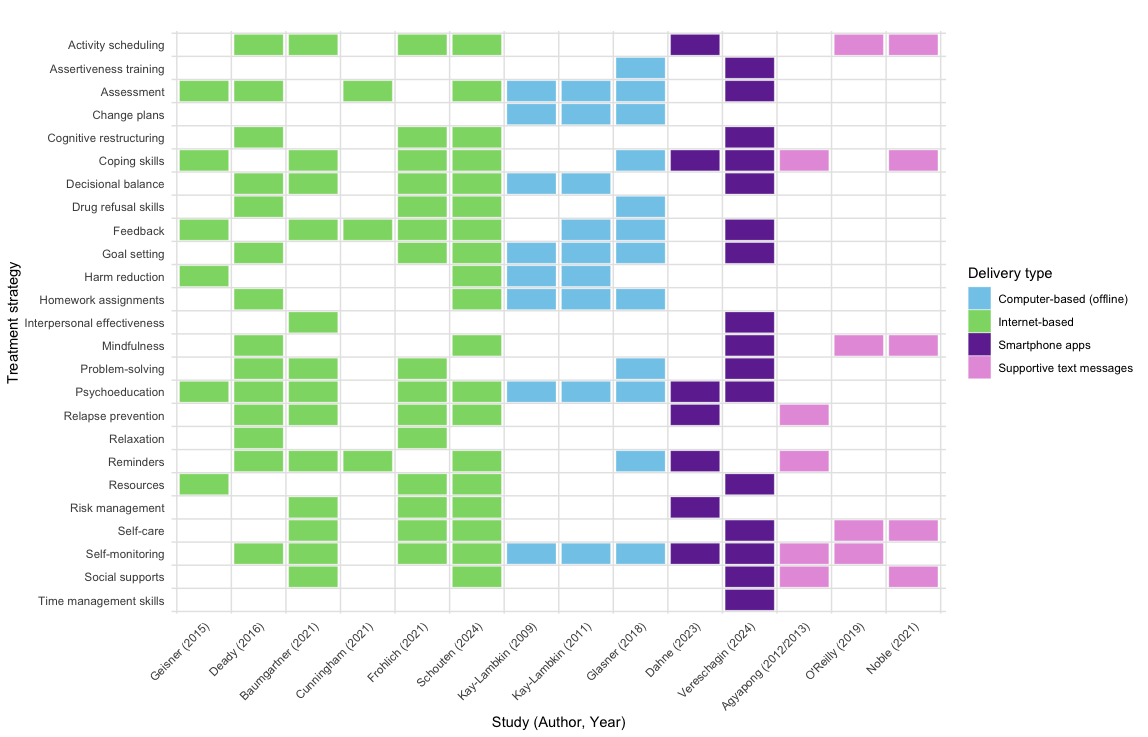


**References**

1. Kay‐Lambkin, F.J., et al., Computer‐based psychological treatment for comorbid depression and problematic alcohol and/or cannabis use: a randomized controlled trial of clinical efficacy. Addiction, 2009. **104**(3): p. 378-388.

2. Kay-Lambkin, F., et al., Acceptability of a clinician-assisted computerized psychological intervention for comorbid mental health and substance use problems: treatment adherence data from a randomized controlled trial. Journal of Medical Internet Research, 2011. **13**(1): p. e1522.

3. Glasner, S., et al., Preliminary outcomes of a computerized CBT/MET intervention for depressed cannabis users in psychiatry care. Cannabis (Research Society on Marijuana), 2018. **1**(2): p. 36.

4. Geisner, I.M., et al., Brief web-based intervention for college students with comorbid risky alcohol use and depressed mood: does it work and for whom? Addictive behaviors, 2015. **42**: p. 36-43.

5. Deady, M., et al., An online intervention for co-occurring depression and problematic alcohol use in young people: primary outcomes from a randomized controlled trial. Journal of medical Internet research, 2016. **18**(3): p. e5178.

6. Baumgartner, C., et al., “Take Care of You”–Efficacy of integrated, minimal-guidance, internet-based self-help for reducing co-occurring alcohol misuse and depression symptoms in adults: Results of a three-arm randomized controlled trial. Drug and alcohol dependence, 2021. **225**: p. 108806.

7. Cunningham, J.A., et al., Randomized controlled trial of online interventions for co-occurring depression and hazardous alcohol consumption: primary outcome results. Internet Interventions, 2021. **26**: p. 100477.

8. Frohlich, J.R., et al., Efficacy of a minimally guided internet treatment for alcohol misuse and emotional problems in young adults: Results of a randomized controlled trial. Addictive behaviors reports, 2021. **14**: p. 100390.

9. Schouten, M.J., et al., Effectiveness of a digital alcohol intervention as an add-on to depression treatment for young adults: results of a pragmatic randomized controlled trial. Psychological Medicine, 2024: p. 1-12.

10. Dahne, J., et al., Behavioral Activation–Based Digital Smoking Cessation Intervention for Individuals With Depressive Symptoms: Randomized Clinical Trial. Journal of Medical Internet Research, 2023. **25**: p. e49809.

11. Vereschagin, M., et al., Effectiveness of the Minder mobile mental health and substance use intervention for university students: randomized controlled trial. Journal of medical internet research, 2024. **26**: p. e54287.

12. Agyapong, V.I., et al., Supportive text messaging for depression and comorbid alcohol use disorder: single-blind randomised trial. Journal of affective disorders, 2012. **141**(2-3): p. 168-176.

13. Agyapong, V.I., D.M. McLoughlin, and C.K. Farren, Six-months outcomes of a randomised trial of supportive text messaging for depression and comorbid alcohol use disorder. Journal of affective disorders, 2013. **151**(1): p. 100-104.

14. O’Reilly, H., et al., Alcohol use disorder and comorbid depression: a randomized controlled trial investigating the effectiveness of supportive text messages in aiding recovery. Alcohol and alcoholism, 2019. **54**(5): p. 551-558.

15. Noble, J.M., et al., Text4Support mobile-based programming for individuals accessing addictions and mental health services—retroactive program analysis at baseline, 12 weeks, and 6 months. Frontiers in psychiatry, 2021. **12**: p. 640795.
